# Involuntary mental health treatment in the era of the United Nations Convention on the Rights of Persons with Disabilities

**DOI:** 10.1371/journal.pmed.1002679

**Published:** 2018-10-18

**Authors:** Faraaz Mahomed, Michael Ashley Stein, Vikram Patel

**Affiliations:** 1 Centre for Applied Legal Studies, University of the Witwatersrand, Johannesburg, South Africa; 2 Harvard Law School Project on Disability, Boston, Massachusetts, United States of America; 3 University of Pretoria Faculty of Law, Centre for Human Rights, Pretoria, South Africa; 4 Department of Global Health and Social Medicine, Harvard Medical School, Boston, Massachusetts, United States of America; 5 Department of Global Health and Population, Harvard TH Chan School of Public Health, Boston, Massachusetts, United States of America

## Abstract

Based on interviews with a variety of participants, Vikram Patel and colleagues advocate for philosophical and practical progress toward recognizing decision-making capacity in people with psychosocial disabilities.

**Summary points**

- The introduction of the United Nations (UN) Convention on the Rights of Persons with Disabilities (CRPD) and General Comment 1 on Article 12 (GC1) has generated considerable debate regarding the assertion that people with psychosocial disabilities (disabilities arising from mental health conditions) always possess decision-making capacity.
- While there is debate over the feasibility and acceptability of supported decision-making, stakeholders interviewed for this study suggested that coercive treatment is heavily overutilized and that there needs to be more dialogue between holders of divergent positions.
- There are a number of important unresolved questions relating to more complex cases and to whether the right guaranteed by the CRPD is absolute and immediate or subject to limitations.
- Ensuring participation of all key stakeholders is essential to realizing the pluralistic vision of the CRPD, as is research focusing on implementation of supported decision-making regimes, particularly in low- and middle-income countries. Training on the provisions of the CRPD is also needed to ensure implementation, and addressing stigmatized beliefs among policy makers about the decision-making and cognitive abilities of mental healthcare users (MHCUs) is critical.

## Introduction

The United Nations (UN) Convention on the Rights of Persons with Disabilities (CRPD) was adopted in 2007 and has since been ratified by 177 countries. It represents a paradigm shift from an impairment-focused, biomedical model of disability to a socially focused, human rights–based model. Impairment arising out of a mental health condition is termed “psychosocial disability” in this model, and laws and clinical protocols governing mental health practice are likely to be informed by the CRPD’s provisions. The Indian Mental Health Care Act of 2017 (MHCA) states that it was drafted because “it is necessary to…harmonize existing laws with [the CRPD]” [1]. Similar processes have taken place or are in motion in 32 countries, [2] illustrating the CRPD’s potential impact on the lives of people living with psychosocial disabilities. This evolution also applies to intellectual disabilities and degenerative conditions (e.g., dementia). Although not covered in detail in this paper, all of these conditions are likely to be affected by the CRPD’s approach to legal capacity, as discussed below.

Biomedical approaches to mental health have allowed for substitute decision-making (i.e., the judgment of a proxy superseding that of an individual when that individual is deemed to be “incapacitated”), leading to considerable maltreatment and abuse of people with psychosocial disabilities [3]. With this history in mind, disability rights advocates, including the World Network of Users and Survivors of Psychiatry (WNUSP), argued strongly for the right to equal recognition before the law, resulting in Article 12 of the CRPD asserting this right. Further interpretation of Article 12 takes the form of General Comment 1 on Article 12 (GC1) [4]. GC1 affirms universal legal capacity (ULC), meaning that all people should be treated as having equal decision-making competency at all times, and mandates a regime of supported decision-making, in which an individual’s will and preference are sought in matters regarding treatment, and assistance is provided to make it possible to ascertain such will and preferences. In the event that this is deemed impossible, the clinician and/or adjudicator is required to apply a standard of the “best interpretation of the individual’s will and preference” instead of the traditional “best interests” principle [5]. The former standard seeks an implicit or explicit communication of the individual’s choices, including through nonverbal communication, while the latter relies on the judgment of an external “trusted” person. This shift is, therefore, a recognition of the equal “personhood” of people with psychosocial disabilities, regardless of cognition [6]. It should be noted that, while the CRPD requires states to adhere to these principles, it does not offer guidance on how they can harmonize their mental health systems with them.

Fiala-Butora and colleagues note that Article 12 was the most heavily contested provision of the CRPD, with numerous states issuing reservations and limitations on its application [7]. A major source of controversy has been the CRPD’s potential impact on the practice of involuntary mental health treatment (including hospitalization and the administration of medication), because ULC essentially renders coercion untenable. Detractors have criticized GC1, arguing that sufficient consultation with clinicians was absent in its formulation [8]. Elsewhere, ULC has been challenged because of ethical difficulties visited upon clinicians who have a duty to protect “vulnerable” mental healthcare users (MHCUs) [9]. Yet others question whether ULC can be realized, because of the difficulty in reaching an interpretation of every MHCU’s preference [10]. Defenders of ULC have suggested that reluctance to relinquish power over MHCUs is a driving factor behind these concerns, noting that ULC is closely associated with other fundamental rights such as dignity and autonomy [11].

Some have called for exceptions that take into account the proportionality of disability and that avoid “serious adverse effects” [12] or address the risk of “imminent and grave harm” [13]. Ironically, it has been noted that these exceptions reawaken the possibility of abuse that the CRPD was intended to prevent [14]. In 2017, the UN Special Rapporteur on the Right to Health called for the “radical reduction and eventual elimination” of coercive treatment, a position that was viewed as insufficient by those seeking abolition without delay [15].

In 2016, the World Psychiatric Association (WPA) issued a Bill of Rights, in which it states.

[w]hen the patient is… incompetent to exercise…judgment…psychiatrists should consult with the family and…seek legal counsel…to safeguard…human dignity and…legal rights [16].

This indicates that the WPA’s thinking may not align with that of the CRPD as expressed in General Comment 1. Noting that a divide exists between disability rights and clinical practice, the World Health Organization (WHO) developed best practice guidelines through the Quality Rights initiative, encouraging health professionals to participate in the implementation of the CRPD, including through application of supported decision-making and offering supports such as training materials to advance this new paradigm [17].

Despite some progress, tensions regarding ULC remain unresolved. This has the potential to hinder progress in implementing reforms and in developing practical solutions. To consider the impact of the debate and key ways in which to advance progress, we sought the perspectives of key stakeholders.

## Stakeholder perspectives

Twelve stakeholders were interviewed in person or over Skype. They included members of the user and survivor movement, representatives of disabled people’s organizations (DPOs) and the UN system, ethicists, clinicians, legal scholars, and policy makers. Using a purposive, deviant sampling method, stakeholders whose positions were known to be diverse based on their publicly held viewpoints were approached [18]. Gender and geographical representivity were also considerations. Several participants held multiple identities (e.g., both clinician and policy maker or both user and DPO representative). The Harvard School of Public Health granted an exemption of Institutional Review Board approval before interviews commenced (IRB17-1943). Coding was undertaken by the interviewer (FM) and a second independent coder (JNB), who was provided with anonymized transcripts. Thematic content analysis occurred initially after seven interviews and then again after a “stopping criterion,” reflecting the decreased probability of retrieving new information, was applied following the 12th interview [19]. Following coding of the first round of interviews, Cronbach’s alpha coefficient was 0.64, rising to 0.82 after the second round, suggesting an acceptable level of inter-rater reliability. More information on the methodology is available in the supplementary files (S1 Text, S1 Table). The results of the thematic analysis are summarized in Table 1.

**Table 1:** Thematic analysis of interview content.

Superordinate themes	Subordinate themes	Key reflections	Exemplary quotes
The complex politics of the debate	“Common ground,” tempered by a lack of trust	Wide agreement that coercive treatment is heavily overutilized	We don’t need to be coercing people…I think we all agree that this shift towards noncoercive treatment is long overdue (Clinician, policy maker, M)
		Questions around the clinical validity of nonconsensual treatment	The [method] of incarcerating people doesn’t…make much clinical sense, apart from its human rights implications (Clinician, policy maker, M)
		Importance of building trust between clinical community and the user and survivor movement	We simply don’t trust each other, and that has a direct impact on progress (Legal scholar, F)
	Power dynamics	Perceived reluctance among psychiatrists to relinquish power	It’s hard to trust [clinicians] because you see things like the [WPA] Bill of Rights, where the first right is the right to access a psychiatrist and the psychiatrist is the gatekeeper for all other rights (User/survivor, F)
		Dominance of discourse and the need for participation	It’s difficult to voice anything [considered to be] capitulating to the clinical community because your allies will disown you…There’s a vocal few and they operate as if they own this narrative (User/survivor, DPO representative, F)
	Plurality and its impact on unity	Heterogeneous views within “sides”	We don’t even agree with each other, let alone the other side (DPO representative, F)
		Tension between the need for “unity” versus the need for participation	In the history of movements, it has been the case that you have people who chain themselves to fences and you have people who engage in negotiation. You need them both (Clinician, DPO representative, M)
Important unresolved questions	Is compromise possible?	Compromise seen by some as a betrayal of the rights won	I don’t think compromise is useful…There needs to be an absolute right (Legal scholar, representative of the UN system, F)
		Compromise may be desirable but is not a necessary condition for progress	We can’t let these individuals derail…progress…Of course their views are relevant…but the reality is that more people are finding ways to compromise (Clinician, representative of the UN system, M)
	Is change progressive or immediate?	Change seen by some as an immediate necessity because the right itself is fundamental to equality	We know these shifts will take time, but that doesn’t change the demand for a fundamental right…Saying that it should be gradual is not what was agreed (Ethicist, legal scholar, M) I think we all know it needs to happen systematically (User/survivor, DPO representative, F)
		Competing perception that “real life is not conducive to absolutes”	It’s clear that health systems need to change…The disagreements and the challenges arise because real life is not conducive to absolutes (Clinician, policy maker, M)
		Potential danger of immediate realization without systemic change and safeguarding	I could see a situation where demanding immediate realization…would result in people being placed in [substandard] services…That [could] actually set back the case for a generation (Legal scholar, M)
	“Hard cases”	Progress on dealing with “hard cases” has not been forthcoming	With the majority of cases, we can agree, but it’s the 1% of outliers that we really just don’t know what to do with (Legal scholar, M)
		Disagreement over exceptions to ULC	If you create an exception, you have undone the fundamental nature of the protection (Legal scholar, F)
The way forward	Innovations in supported decision-making and the need for more research	Important developments in supported decision-making, such as advance directives, open dialogue, and personal ombuds	There are some really fantastic achievements, which shouldn’t be discounted (Clinician and policy maker, M)
		Research is needed to develop best practice in supported decision-making	I think we still have a lot to learn about how to actually implement supported decision-making (Representative of the UN system, M)
		Supported decision-making innovations have concentrated on high-income countries, where resource availability is less constrained	[The personal ombud] works in Scandinavia where they have a lot more resources than we have, but we need to find ways of doing things here that are practical (Policy maker, M)
		Conceptual research is also needed on the “best interpretation of will and preference” standard	What tools can be developed to engage with the [MHCU’s] will and preference? How can will and preference be protected from undue influence? (Legal scholar, M)
	The need for multidisciplinary “safe spaces” for dialogue	These spaces are being developed and need broad-based participation	This conversation is taking place on two planes, the legal and policy plane and the psychiatry plane (Clinician, policy maker, M) The lawyers think of this as a conceptual issue and don’t engage with the practical. The practitioners see this as a clinical issue but haven’t engaged [with law and policy] (DPO representative, F)
		Participation of clinicians is needed	There seems to be little incentive for clinicians to participate…it makes sense because you know that if you [participate], people are going to say you’re a human rights violator (Legal scholar, M)
	Training and sensitization within policy spaces to implement the new paradigm	Lack of interest/understanding of the CRPD in many policy spaces	In some places, they have been talking for years…In others, the people responsible for implementation don’t know what they are supposed to be enforcing (Policy maker, M)
		Stigma relating to mental health is a barrier to engaging with systemic change in mental health systems	It doesn’t matter if we change how we think and others are still thinking the way they think (User/survivor, DPO representative, M)

Abbreviations: DPO, disabled people’s organization; F, female; M, male; MHCU, mental healthcare user; ULC, universal legal capacity; UN, United Nations; WPA, World Psychiatric Association.

### The complex politics of the debate

A universally held position among interviewees was that involuntary treatment is heavily overutilized, bringing into question both its clinical and ethical validity. Trust between the “sides” (Clinician, policy maker, M) was a major challenge. Historically, the relationship between psychiatrists and MHCUs has been fraught. There would need to be continued dialogue, but some also raised the possibility of more formal “truth and reconciliation” processes.

Some stakeholders believed that a desire persisted among clinicians to maintain power over MHCUs, while power dynamics were also a source of conflict within the user and survivor movement. A variety of perspectives is evident within the psychiatric community, with moderate actors more inclined to seek ways of adapting practice to new realities and “extreme” voices, who are resistant to change. Similarly, “fundamentalism” (User/survivor, F) within some segments of the user and survivor movement was a perceived obstacle because some actors treated ULC as an absolute, although others argued that moderation was problematic precisely because of the absolute nature of the right. While “unity” may be desirable for some, it is not a necessary condition for progress to occur, particularly because there is a significant need for “more opinions on such a new debate” (Clinician, DPO representative, M) and because the CRPD advocates maximizing participation.

### Important unresolved questions

Many speakers viewed ULC as fundamental and so were of the view that Article 12 rights could not be compromised. Similarly, this “impasse” (Representative of the UN system, M) is both about whether the CRPD’s provisions can be realized and whether such realization is progressive or immediate. A gradual shift to supported decision-making is seen as an important step by some but, for others, this would be insufficient.

A major challenge remains “hard cases,” for instance, when an individual is comatose, when there is a significant risk of the individual causing harm to him- or herself or to others, or when there may be cognitive impairment or substance use affecting decision-making capacity. The possibility of exceptions in such instances was a contentious issue, with some participants noting that the progress made under the CRPD should not be “endanger[ed]” by leaving open a “loophole” (User/survivor, F). By contrast, it was viewed as “unreasonable” to suggest that no involuntary treatment could ever occur because, as one clinician put it, “real life is not conducive to absolutes” (Clinician, policy maker, M). In the opinion of the stakeholders interviewed, neither clinical protocols nor legislative spaces have yielded progress insofar as “hard cases” are concerned, meaning that little practical guidance on the application (or lack thereof) of the CRPD’s provisions in situations of emergency or other “hard cases” exists.

### The way forward

Important developments were noted, such as the Quality Rights tool kit, which provides training materials for capacitating clinicians on the provisions of the CRPD and use of instruments such as advance directives. However, stakeholders were of the view that resource differentials necessitate a contextual approach, and there is a need for more research on supported decision-making models that can be applied to low-resource settings. Similarly, they felt that other areas such as the “will and preference” standard also require further theorizing, as there is very little conceptual or jurisprudential discourse on this approach.

While dialogue was accepted to be a common goal, there were divergent epistemological positions informed by different disciplines. According to stakeholders, the positivist clinical perspective and the interpretivist legal and policy lens have not engaged substantially with each other. Moves to create interdisciplinary “safe and neutral spaces” (Clinician, DPO representative, M) were said to require substantial support. This refers to spaces for the exchange of ideas regarding ULC and the practical implications of its introduction that eschew ideology and that explicitly seek to be respectful of all opinions and positions. According to some interviewees, increased participation in these discussions, particularly among clinicians, is also necessary to engage with the subject of how to implement the CRPD in practice.

Considerable levels of stigma regarding the abilities and rights of people with psychosocial disabilities, even among policy makers and clinicians, and lack of awareness of the CRPD were also mentioned as obstacles to reforms of mental health systems, because they render it impossible to engage meaningfully and respectfully in debates such as that on ULC. Ultimately, some stakeholders felt that addressing these obstacles through efforts aimed at training and sensitization will be necessary to translate the ideological goals of the CRPD into the lived experience of change.

## Discussion

The results highlighted important political and epistemological differences that impacted how the debate on legal capacity has been evolving, as well as unresolved questions around the practical application of ULC and key steps that should be taken to develop this discourse further. There appears to be broad agreement among the stakeholders interviewed that involuntary mental health treatment is heavily overutilized. Therefore, calls for “radical reduction” in nonconsensual methods, while perhaps not sufficient for some, are likely to have widespread support. This already provides a basis for important changes to be made.

Training on the CRPD should be undertaken with policy makers to engage meaningfully with any potential reforms, while stigma reduction among policy makers to develop awareness of the needs and rights of MHCUs can also foster more robust efforts to realize the rights of people with psychosocial disabilities. Multidisciplinary dialogue, making use of the “safe and neutral spaces” alluded to, can be a key catalyst for progress. The suggestion that “more opinions” are needed “on such a new debate” is an important indication of the need for participatory conversations around ULC and should be heeded in ways that are pluralistic and prioritize the voices of MHCUs. Participation and pluralism are important cornerstones of the CRPD. Where disagreement appears challenging is in the heavily contested nature of the debate and the complex power dynamics alluded to earlier.

Further engagement is needed to consider the nature and scope of the right to equal recognition before the law, because there remains a lack of clarity as to whether this right is an absolute one and because there are differing implications if the right is progressive or immediately realizable (i.e., immediate realization requires more radical and urgent change, while progressive realization is likely to be predicated on factors such as the availability of resources and an examination of the specific vulnerabilities of each group of MHCUs). The fact that GC1 focused only on normative guidance without considering practical application appears to have left substantial ambiguity in its interpretation. This has contributed to difficulties for clinicians, who must make some difficult decisions regarding MHCUs who may pose a danger to themselves or others or who may be vulnerable to abuse, exploitation, or neglect if they do not receive the treatment that practitioners have been trained to administer (i.e., the “hard cases”). Addressing these questions of clinical practice will require further engagement that can benefit from the input of MHCUs themselves and that recognizes the evolving thinking in relation to “personhood,” which posits that clinicians are no longer expected or warranted to be arbiters of the best interests of those in their care.

Legal and conceptual thinking will need to focus on developing the “best interpretation of the individual’s will and preference” standard. Similarly, legislators have struggled to find a balance between the CRPD approach and established practice in the development of new laws. In 2016, Costa Rica abolished guardianship, creating the legal figure of “guarantor for equality before the law” [20]. Peru’s General Law on Disability reasserts ULC, while leaving decision-making regimes to the civil code, which at the time of writing is unpublished [21]. The Indian MHCA institutes advance directives, although they apply when an individual has “ceased” to have capacity and may be revoked “at any time” according to the Act, suggesting that an individual may change their mind, potentially as a result of their mental health condition, and do not apply at all in emergency situations [1]. An example of “fusion” legislation [22], the Northern Irish Mental Capacity Act of 2016 provides a single framework governing the treatment of any person deemed to lack capacity. It provides for substitute decision-making but requires that a supporter pay “special regard” to the individual’s wishes [23]. These provisions have clearly sought balance, but they also demonstrate how elusive balance might be. More refinement is needed, and steps should be taken to develop supported decision-making systems that respect autonomy and dignity but also contain safeguards to prevent abuse, coercion, or exploitation.

Efforts to engage with supported decision-making have been gaining traction. Advance directives predate the CRPD in some contexts [24]. Peer support models, such as the “circle of support,” bringing together supporters of MHCUs to discuss their will and preferences, have been utilized [25]. The process of “open dialogue,” whereby discussion is generated in family and treatment systems, has been proposed [26], while the introduction of a personal ombud for MHCUs in Sweden has also demonstrated potential [27]. Even so, implementation requires adaptability for contexts in which resources are limited. Research is needed to develop best practices in culturally and economically diverse contexts. A 2012 review found that supported decision-making provisions were particularly wanting in low- and middle-income countries [28], despite isolated efforts such as the “circle of care” implemented in India [29]. The UN Special Rapporteur on the Rights of People with Disabilities reported in 2017 that research projects were underway in 17 countries on six continents [30]. Considering the crucial need for locally developed solutions, this should be supported. Similarly, efforts to engage with MHCUs directly on their preferences regarding supported decision-making through means such as surveys and social media can contribute significantly to this discourse.

This paper suggests that important progress has been made, but more is needed to actualize the rights of people living with psychosocial disabilities, including constructive debate among those with divergent positions and research to establish best practice in supported decision-making, especially in resource-challenged settings. Addressing stigmatized beliefs and attitudes among policy makers relating to the decision-making abilities of people with psychosocial disabilities is another important avenue through which progress can be fostered, and ensuring the participation of MHCUs, particularly from low- and middle-income countries, in these conversations is essential. It is time that mental health systems around the world heed the calls of various stakeholders to transform, and hopefully the actions outlined above can aid in such transformation.

## Supporting information

S1 Text Methods. (DOCX)

S1 Table Sampling summary. (DOCX)

## Abbreviations

CRPD: Convention on the Rights of Persons with Disabilities
DPO: disabled people’s organization
GC1: General Comment 1 on Article 12
MHCA: Mental Health Care Act of 2017
MHCU: mental healthcare user
ULC: universal legal capacity
UN: United Nations
WHO: World Health Organization
WNUSP: World Network of Users and Survivors of Psychiatry
WPA: World Psychiatric Association
