# Supplementary material for: Involuntary mental health treatment in the era of the United Nations Convention on the Rights of Persons with Disabilities
Source: PLoS Med. 2018 Oct 18;15(10):e1002679. doi: 10.1371/journal.pmed.1002679 (PMC6193619; doi:10.1371/journal.pmed.1002679)
Supplement: S1 Text — (DOCX) [file pmed.1002679.s001.docx]

S1 Table. Sampling Summary

| N (total) | 12 | |
| --- | --- | --- |
| Gender | Male | 6 |
|  | Female | 6 |
| Identity | User/survivor | 6 |
|  | Policy-maker | 6 |
|  | Clinician | 5 |
|  | Legal scholar | 4 |
|  | Ethicist | 2 |
|  | Representative of a Disabled People’s Organisation | 2 |
|  | Representative of the UN system | 2 |
| Geographical location | North America | 2 |
|  | South America | 1 |
|  | Sub-Saharan Africa | 3 |
|  | Europe | 3 |
|  | Asia | 2 |
|  | Australia/Oceania | 1 |
