# Supplementary material for: Involuntary mental health treatment in the era of the United Nations Convention on the Rights of Persons with Disabilities
Source: PLoS Med. 2018 Oct 18;15(10):e1002679. doi: 10.1371/journal.pmed.1002679 (PMC6193619; doi:10.1371/journal.pmed.1002679)
Supplement: S1 Table — (DOCX) [file pmed.1002679.s002.docx]

S1 Text. Methods

- 1. Data Collection

To examine the opinions and experiences of various stakeholders in the debate regarding legal capacity for people living with psychosocial disabilities, paying particular attention to the issue of involuntary treatment, key informant interviews, using a semi-structured interview protocol, were chosen as the method of data collection. This is because there were numerous varying viewpoints that required in-depth examination to capture the nuances in what appeared to be a spectrum of positions.

The study was conducted through in-person interviews and through Skype interviews when participants were in geographical locations not physically accessible to the researcher. Interviews considered the experiences of policy-makers, clinicians, legal and public health scholars and mental health and disability rights advocates (including those from the user and survivor communities) – collectively referred to as stakeholders - in implementing, advocating for or studying issues such as legal capacity under the CRPD, the supported decision-making paradigm, and clinical practice and legislation and policy development in light of the CRPD’s position on legal capacity. Interviews were conducted between January and March 2018 in person or over Skype. They ranged between 30-60 minutes in length and were audio recorded with the consent of participants.

- 1. Sample

Sampling of key informants was purposive in nature, with the express purpose of seeking out a plurality of views on the subject matter such that a broad spectrum of positions could be highlighted and examined. A deviant sampling method was employed to capture those views that were most characteristic of the diversity in the debate. This meant engaging with policy-makers, academics, clinicians and disability rights advocates, including mental health care users themselves, whose positions were known to be diverse based not only on their professional orientation but, also, on their publicly held viewpoints and previous statements on the subject. Gender and geographical representivity were also key concerns, as evidenced by Table 1 below. As such, sampling for the study was guided by Morse’s (1995) principle of ‘maximum variation’ and by what Burmeister and Aitken (2012) refer to as ‘critical depth’ in the pursuit of views and ideas.

Guided by our own time and resource constraints, and following from Francis et al.’s (2009) methodological guidance regarding interview-based research, an initial analysis sample of 7 key informants was constructed. Following the initial analysis, further interviewees were identified to capture the principles of ‘maximum variation’ and ‘critical depth’. Furthermore, Francis et al. (2009) recommend the application of a ‘stopping criterion’ for interviews, guided by the probability of new evidence emerging and ceasing to conduct further collection of data when this evidence is not forthcoming. In this instance, the ‘stopping criterion’ was applied after 12 interviews as it became clear that no new evidence had emerged in several preceding interviews. This process is considered akin to the more traditionally-accepted concept of data saturation, where no new information is likely to be gleaned from continuing data collection and when there is sufficient richness in the data to meet the initial aims of the study and to answer the research questions.

- 1. Data Analysis

Interviews were recorded for accuracy and transcribed verbatim. Recognizing that there were some key themes already emerging from the literature (such as divergent discourses between the biomedical and the rights-based paradigm and the challenge of implementing supported decision-making in practice), a thematic analysis that accommodated these themes was chosen as the method of analysis. Coding was undertaken by the interviewer as well a second independent coder, who was provided with anonymized transcripts and a process of discussion was undertaken where any disagreements were noted. The process of coding was done manually. Cronbach’s alpha was calculated to examine convergence between coders at each iteration of coding.

Interviews were organized using Labov’s (1972) thematic organization methodology, which prioritizes meta-themes and an exploration of manifest as well as latent content. Following completion of the ‘initial analysis’ phase of interviews, data from these interviews were ‘live coded’ into *a priori* and emergent themes (Labov, 1972). Thomas (2003) notes that research relating to the health and social sciences lends itself well to an approach that is determined by the research objectives (deductive) and from what emerges from raw data (inductive). He suggests a ‘generalist’ inductive approach that condenses extensive and varied raw data into a brief, summary format, establishes clear links between the research objectives and the summary findings derived from the raw data and develops a model about the underlying structure of experiences and processes evident in the dataset (Thomas, 2003).

The structural framework was iterative in nature, shaping and reshaping as new data emerged and new experiences were added to the universe of data (Thomas, 2003). This process took place initially after the seventh interview. Following coding of the first round of interviews, Cronbach’s alpha coefficient was 0.64. After completion of the second round of interviews, and once the ‘stopping criterion’ had been applied, another iteration of coding took place, examining the totality of the data as recommended by Francis et al. (2009) and seeking to construct the model of an underlying structure that Thomas (2003) refers to. Cronbach’s alpha rose to 0.82 in this instance, suggesting an acceptable level of inter-rater reliability.

Three super-ordinate themes were arrived at to construct this underlying structure, namely:

1. The complex politics of the debate
2. Important unresolved questions
3. The way forward

Subordinate themes were also identified within this framework, and these are outlined in the results section.

- 1. Ethics

The sample for this study consisted of people who have been known to hold particular viewpoints and positions and who have experience in engaging with the subject matter at hand. The Harvard School of Public Health, therefore, granted an exemption of Institutional Review Board approval before interviews commenced (IRB17-1943).

References:

Burmeister E & Aitken LM. (2012). Sample size: How many is enough? Australian Critical Care, 25, 4, 271.

Francis JJ et al. (2010). What is an adequate sample size? Operationalising data saturation for theory-based interview studies. Psychology & Health, 25, 10, 1229.

Labov W. (1972). Some principles of linguistic methodology. Language in Society, 1, 97.

Morse JM. (1995). The Significance of Saturation. Qualitative Health Research, 5, 2, 147.

Thomas DR. (2003). A general inductive approach for qualitative data analysis. http://frankumstein.com/PDF/Psychology/Inductive%20Content%20Analysis.pdf
